# An Unusual Salivary Gland Tumor Mimicking Papillary Thyroid Carcinoma: Mammary Analog Secretory Carcinoma

**DOI:** 10.3389/fendo.2018.00555

**Published:** 2018-09-25

**Authors:** Sylvia L. Asa, Ozgur Mete

**Affiliations:** ^1^Department of Pathology, University Health Network, Toronto, ON, Canada; ^2^Department of Laboratory Medicine and Pathobiology, University of Toronto, Toronto, ON, Canada

**Keywords:** thyroid, salivary gland, mammary analog secretory carcinoma, papillary thyroid cancer, immunohistochemistry

## Abstract

Thyroid tumors usually present as masses in the thyroid gland. While the majority of these tumors represent neoplasms of thyroid tissues, mainly of follicular epithelial cell differentiation, the differential diagnosis includes other lesions, such as C cell neoplasms (medullary thyroid carcinoma), intrathyroidal parathyroid, or thymic tumors, soft tissue tumors, and hematologic neoplasms as well as metastatic malignancies. Rare tumors are of salivary gland types. This case illustrates an unusual tumor of salivary gland type, an intrathyroidal mammary analog secretory carcinoma (MASC). The pathogenesis, diagnostic pitfalls, and therapeutic implications of this unusual tumor are discussed.

## Background

Thyroid tumors are common and the vast majority represent tumors derived from follicular epithelial cells (1). Most are differentiated thyroid neoplasms, including follicular adenomas, well differentiated follicular tumors with low metastatic potential (including NIFT-P, encapsulated follicular variant papillary thyroid carcinoma with minimal capsular invasion and minimally invasive follicular carcinomas), and classical papillary carcinomas. Rare aggressive follicular carcinomas or follicular variant papillary carcinomas show widespread capsular invasion or angioinvasion and some tumors progress with loss of differentiation, including poorly differentiated carcinomas and anaplastic carcinomas.

**Figure 1:**
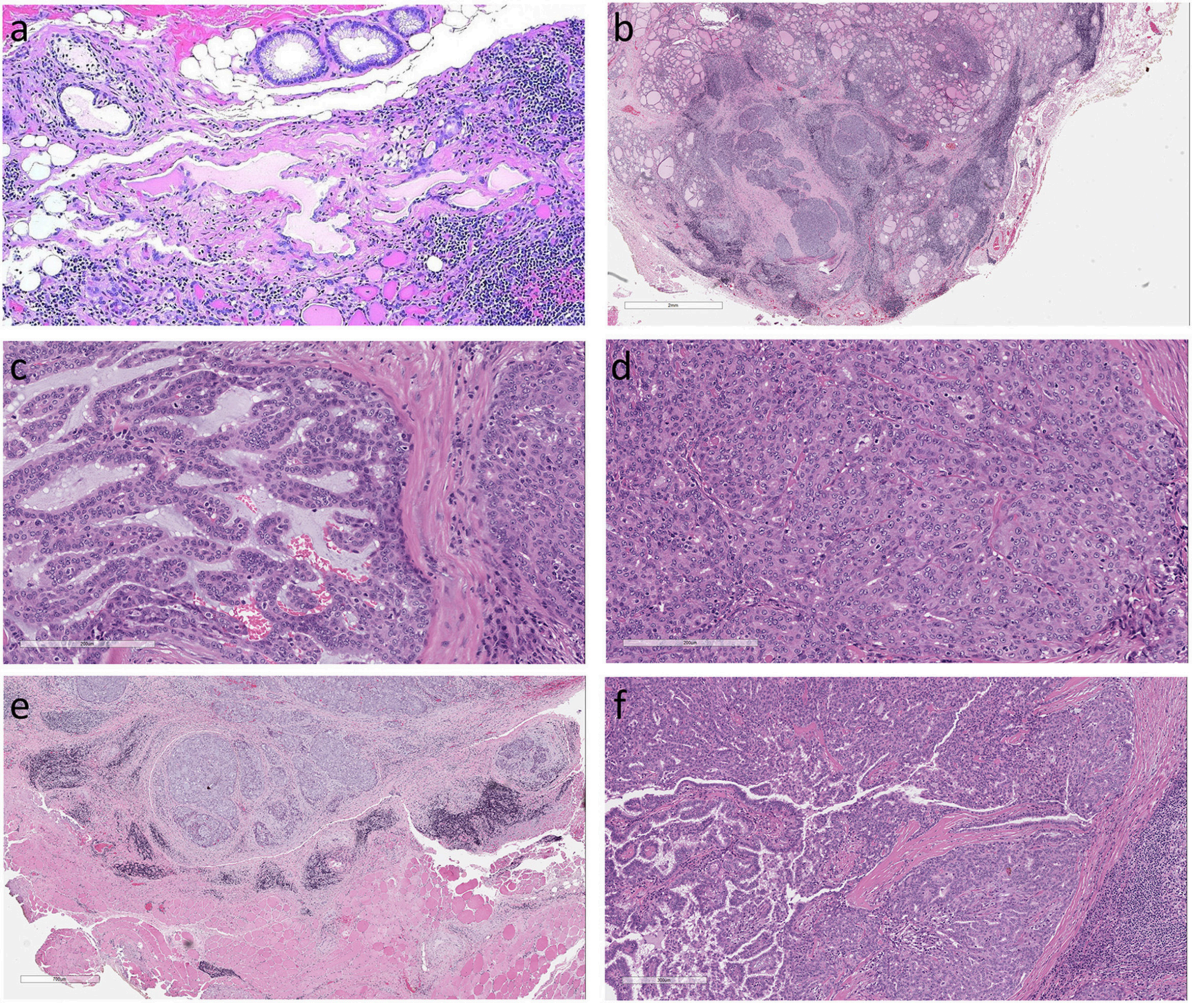
Histologic features of Mammary Analog Secretory Carcinoma of Thyroid **(a)**. The origin of this unusual tumor is unknown but may be from incidental intrathyroidal salivary gland rests as seen in this normal thyroid (not from the patient reported) **(b)**. The thyroid tumor in the case described is an infiltrative tumor composed of solid sheets and nests of epithelial cells in a fibrous stroma. The surrounding thyroid exhibits chronic lymphocytic thyroiditis **(c)**. The solid sheets were punctuated by small cribriform areas and microcysts with pseudopapillae and a few true papillae with fibrovascular cores **(d)**. The homogeneous tumor cells had abundant cytoplasm and monotonous round nuclei with clear nucleoplasm and conspicuous large nucleoli but no indentations or inclusions **(e)**. There was extrathyroidal extension into surrounding skeletal muscle **(f)**. In one area of the tumor there was a small 0.2 cm focus of classical papillary microcarcinoma.

Other thyroid tumors include medullary carcinomas of C cell differentiation and tumors that are composed of cells that are not unique to the thyroid (1). These include intrathyroidal parathyroid lesions and thymic tumors that are explained by their embryologic origin and locations near and occasionally within the thyroid. Soft tissue tumors can arise from soft tissue components of the thyroid region. Primary thyroid lymphomas and other hematologic neoplasms can present as thyroid masses and some thyroid nodules represent metastatic malignancies. Other unusual thyroid tumors can be of salivary gland types, and these are explained by the occasional finding of salivary gland tissue within the thyroid (Figure 1a).

This report describes a case of a salivary gland type of tumor that can be confused with a papillary thyroid carcinoma. The clinical and therapeutic implications of the diagnosis are discussed.

## Case report

A 72 year old woman was found to have a 2.5 cm nodule in the left thyroid. Thyroid function tests were within the normal range. She had no family history of thyroid or other endocrine disease. Her medical history was unremarkable. A fine needle biopsy of the lesion was diagnosed as “suspicious for neoplasm.” She underwent left hemithyroidectomy.

The tumor was diagnosed as papillary thyroid carcinoma by the pathologist at the originating institution. There was extrathyroidal extension. A consultation from a thyroid expert confirmed the diagnosis. The patient was referred to our institution for completion thyroidectomy and radioactive iodine therapy. Pathology review was requested.

The patient was evaluated for metastatic disease and none was identified. She is alive and well with no evidence of recurrence 18 months later. The patient provided informed signed consent for publication of her data.

### Pathology findings

The thyroid contained an infiltrative tumor that had areas of follicular and papillary architecture but the overall morphology and cytologic features were atypical for a tumor of thyroid follicular differentiation. The surrounding thyroid exhibited chronic lymphocytic thyroiditis. The tumor was composed of solid sheets and nests in a fibrovascular stroma (Figure 1b) with cribriform areas, microcysts, cleft-like structures, and focal pseudopapillae with a few true papillae (Figure 1c). The tumor cells were relatively homogeneous with abundant eosinophilic cytoplasm and monotonous round nuclei with clear nucleoplasm and conspicuous large nucleoli but no indentations or inclusions (Figure 1d). There was frank extrathyroidal extension into surrounding skeletal muscle (Figure 1e). In one area of the tumor there was a small 0.2 cm focus of classical papillary microcarcinoma with the distinctive features of that entity that were clearly different from the rest of the lesion (Figure 1f).

**Figure 2:**
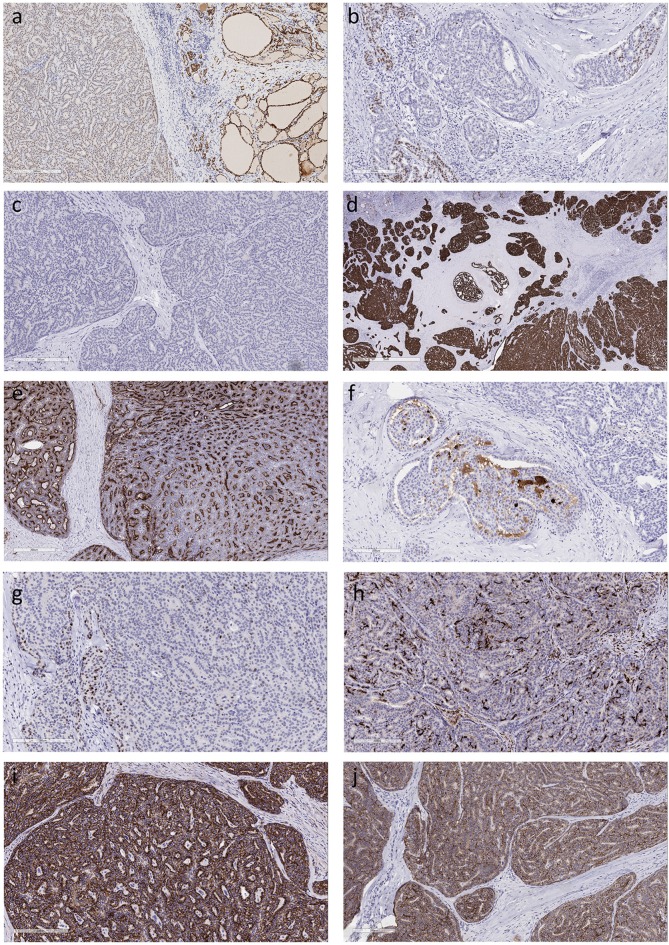
Immunohistochemical features of Mammary Analog Secretory Carcinoma of Thyroid **(a)**. The tumor cells exhibit diffuse positivity for monoclonal PAX-8 that is much weaker than in the surrounding thyroid **(b)**. There is very focal positivity for TTF-1 (clone: SPT24); some of the stained cells might be entrapped follicular epithelial cells **(c)**. The tumor cells are completely negative for thyroglobulin **(d)**. The tumor exhibits strong diffuse positivity for cytokeratin 19 **(e)**. Staining for CEA with a polyclonal antiserum yields diffuse reactivity, however a monoclonal CEA antibody resulted in a completely negative stain **(f)**. Tumor cells are positive for gross cystic disease fluid protein-15 **(g)**. Scattered tumor cells express p63 **(h)**. Dendritic type cells that are strongly positive for S100 protein are distributed throughout the tumor **(i)**. Beta-catenin staining is intact at the cell membrane and there is no nuclear translocation **(j)**. Positivity for E-cadherin is retained at the cell borders.

Immunohistochemistry of the dominant tumor identified diffuse but weak monoclonal PAX-8 nuclear reactivity (Figure 2a) but TTF-1 (clone: SPT24) was only focal and weak (Figure 2b) and thyroglobulin staining was completely negative (Figure 2c). Stains for Cytokeratin 7 and Cytokeratin 19 (Figure 2d) were diffusely positive but Cytokeratin 5 was only focally expressed. Although polyclonal CEA was positive (Figure 2e), monoclonal CEA was negative, as was synaptophysin and chromogranin-A. Scattered tumor cells were positive for gross cystic disease fluid protein-15 (GCDFP-15) (Figure 2f), some stained for p63 (Figure 2g) and stellate cells were identified by localization of S100 protein (Figure 2h). CD5 positivity was restricted to infiltrating lymphocytes. Beta-catenin (Figure 2i) and E-cadherin (Figure 2j) positivity was intact at the tumor cell membrane and there was no nuclear translocation.

The diagnosis was changed to Mammary Analog Secretory Carcinoma (MASC), an unusual tumor of salivary gland, associated with a 0.2 cm papillary microcarcinoma.

## Discussion

Mammary analog secretory carcinoma (MASC) has been recently identified as a unique salivary gland tumor that occurs mainly in the parotid but can also occur in minor salivary glands (2). It has also been reported in the sinonasal tract, lip, skin, thyroid gland, and lung (3–5). The thyroid gland is known to be a site of mucoepidermoid carcinoma and other lesions of salivary gland types, so it is not surprising that it has also been found to harbor MASC (4, 6–9).

A characteristic finding in the initial molecular studies of MASC was the presence of *t(12;15)(p13;q25)* resulting in *ETV6-NTRK3* translocation. This was reported to be specific for this tumor type and was not documented in other salivary gland tumors. However, a recent report of 10 cases with typical morphology and immunoprofile identified that some such tumors harbor a novel *ETV6-RET* translocation (10). It is interesting to speculate on the possible cytogenetic and molecular relationships between these tumors and carcinomas of follicular epithelial differentiation that also harbor rearrangements of *RET* and *NTRK* (11).

The diagnosis of MASC in thyroid can be challenging as illustrated in this case. Many features mimic papillary thyroid carcinoma. The cytology can be misdiagnosed (8, 9). The architecture can resemble that of papillary thyroid carcinoma and the diffuse expression for PAX-8 can result in a misdiagnosis, however, the staining for the biomarkers of thyroid differentiation, when present, is weak and focal (7) as in our case. In addition, papillary thyroid carcinomas may exhibit diffuse positivity for cytokeratin 19 (12), again leading to a potential for misdiagnosis. However, differentiated thyroid carcinomas are rarely if ever completely negative for thyroglobulin, and the lack of positivity should raise the possibility that this is not a primary tumor of thyroid follicular cell derivation, especially when the tumor is not diffusely positive for TTF-1 and PAX8. TTF-1 (clone 8G7G3/1) has been reported to be negative in MASC (13) whereas we found very focal TTF-1 reactivity in our case using the SPT24 clone; while some of the stained cells may be entrapped follicular epithelial cells, it is possible that there is clone-dependent focal expression for TTF-1 in these tumors. The identification of basophilic/blue luminal secretory material and numerous S100-positive cells should prompt consideration of the diagnosis of MASC that can then be confirmed by the identification of mammary markers such as GCDFP-15, mammoglobin (2) or GATA-3 (6).

The distinction of MASC from papillary carcinoma can be complicated by the presence of a true papillary carcinoma. Two previous cases have been reported to have a minor component of papillary thyroid carcinoma (13) and similar to our case, the two lesions had distinct morphologic and immunophenotypic features. While the *ETV6-NTRK3* translocation has been described in both tumors, and the two tumor types may indeed be causally related, it remains to be determined whether these represent collision tumors or divergent differentiation (clonal trans-differentiation).

MASC can be a very aggressive tumor; most reported cases have extensive local invasion and lymph node metastases and some have pursued an aggressive clinical course with the development of distant metastases. It is important to ensure the correct diagnosis; several cases have been misdiagnosed as papillary thyroid carcinoma but were not responsive to the usual therapy for that disease, radioactive iodine (6, 13). Our case was referred for radioactive iodine therapy based on a similar misdiagnosis. These tumors require other management approaches, including possible response to entrectinib (a tyrosine kinase inhibitor targeting TrkA, TrkB, TrkC, ROS1, and ALK), however the development of resistance has been reported (14).

## Concluding remarks

This case illustrates an unusual tumor of salivary gland type, an intrathyroidal MASC. The occurrence of salivary gland tumors in the thyroid is unusual and they can mimic primary thyroid carcinomas, however the distinction of these tumors from neoplasms of thyroid follicular epithelium is important, since the treatment approaches are distinct. The potential association of mammary analog secretory carcinoma with papillary thyroid carcinoma raises important questions about possible common precursor cells. Both tumor types have common gene fusions, pointing to possible similar pathogenetic mechanisms.

## Author contributions

SA and OM case review. SA writing and image preparation. OM review and editing of manuscript.

### Conflict of interest statement

The authors declare that the research was conducted in the absence of any commercial or financial relationships that could be construed as a potential conflict of interest.
